# Immunoreactive Cells After Cerebral Ischemia

**DOI:** 10.3389/fimmu.2019.02781

**Published:** 2019-11-26

**Authors:** Yijie Wang, John H. Zhang, Jifang Sheng, Anwen Shao

**Affiliations:** ^1^State Key Laboratory for Diagnosis and Treatment of Infectious Diseases, Collaborative Innovation Center for Diagnosis and Treatment of Infectious Diseases, First Affiliated Hospital, College of Medicine, Zhejiang University, Hangzhou, China; ^2^Department of Physiology and Pharmacology, Loma Linda University School of Medicine, Loma Linda, CA, United States; ^3^Department of Neurosurgery, Second Affiliated Hospital, School of Medicine, Zhejiang University, Hangzhou, China

**Keywords:** immunity, ischemia, immunomodulation, stroke, cells, review

## Abstract

The immune system is rapidly activated after ischemic stroke. As immune cells migrate and infiltrate across the blood-brain barrier into the ischemic region, a cascade of cellular and molecular biological reactions occur, involving migrated immune cells, resident glial cells, and the vascular endothelium. These events regulate infarction evolution and thus influence the outcome of ischemic stroke. Most immune cells exert dual effects on cerebral ischemia, and some crucial cells may become central targets in ischemic stroke treatment and rehabilitation.

## Introduction

Stroke has high morbidity and mortality rates around the world and became the leading cause of death in China in 2017 (1). It accounts for about 1 in 19 deaths in the U.S. (2). Ischemic stroke is associated with considerable morbidity and mortality in eastern and northern Asia (3). In recent decades, understanding of the mechanism, diagnosis, and therapy of ischemic stroke has improved. Patients with stroke experience neuronal damage and functional deficits, which are tightly connected with immune responses during and after ischemia. Reduced focal cerebral blood flow induces a series of metabolic, neurologic, and immunologic reactions, which are followed by neuronal cell death (4). Studies of immune responses during and after ischemic brain damage have revealed some probable pathways of post-ischemic cerebral injury, and will be meaningful in identifying potential therapies for ischemic stroke. It is crucial to further investigate the mechanical processes of the immunological and inflammatory reactions after cerebral ischemia. Immune responses implicate the blood-brain barrier (BBB); vascular endothelial cells; glial cells; inflammatory mediators; and immune cell infiltration, migration, and activation. Immune cells are rapidly activated and recruited to the stroke site (5), where they continue to affect infarction progression and prognosis. This article reviews the mediators, regulatory factors, and interaction behaviors of different subpopulations of both brain resident and peripheral immune cells during the immune process after cerebral ischemia.

## Leukocytes In Post-Ischemic Immune Responses

Due to increased BBB permeability and compromised BBB integrity in the acute phase after stroke (6–8), leukocytes aggregate in the ischemic region of middle cerebral artery occlusion (MCAO) models as early as 30 min after occlusion (9). Mediated by leukocyte-endothelial cell interactions on the endothelial vascular walls, leukocyte infiltration influences the evolution and outcome of ischemic injury.

### Neutrophils

Infiltration of polymorphonuclear neutrophils (PMNs) in the acute stage is a feature of post-ischemic inflammatory responses (10, 11). Studies on the role of PMNs in the cerebral post-ischemic immune response suggest that neutrophils aggravate the prognosis after stroke (12). Neutrophil infiltration is responsible for increased recruitment of other immune cells, and thus induces a complicated series of effects on ischemic damage (13). Although there are numerous studies supporting the long-believed detrimental effects that neutrophil infiltration into the infarct region causes in the acute stage of stroke (14–16), evidence also suggests that neutrophils contribute to beneficial regulation processes.

Neutrophils are involved in tissue remodeling after stroke (17). Matrix metalloproteinases (MMPs) are a family of secreted and membrane-bound proteases that influence extracellular matrix (ECM) and tissue repair processes (18, 19). They are usually crucial in leukocyte recruitment. Various MMP subtypes have been studied. For example, MMP-9 promotes BBB leakage, neuronal cell death, and hemorrhage in the early phase after stroke, while it later mediates brain regeneration and neurovascular remodeling (17, 20). High MMP-2 levels in an ischemic stroke patient is considered to suggest a stable or recovering condition, while MMP-9 suggests worse prognosis (21). MMP-2 is considered to have beneficial influences on post-ischemic immune responses (22). In general, MMPs have dual effects. Neutrophils produce and release substances like vascular endothelial growth factor (VEGF) and transforming growth factor-β (TGF-β), thus regulating various immune regulation activities (6). Neutrophils are also involved in clearance of dead cells, debris, and bacteria, creating a more suitable microenvironment for repair and recovery (6, 13, 23). Neutrophil extracellular traps (NETs), the structural fibers produced by neutrophils, are composed of granule and nuclear constituents. As a conserved innate antimicrobial strategy, NET release has been reported to play a role in extracellular bactericidal activities (24).

Many pro-inflammatory mediators can induce NET release during inflammatory processes, including interleukin-8 (IL-8), tumor necrosis factor-α (TNF-α), and platelet-activating factors (11). Non-inflammatory factors such as amyloid fibrils also trigger NETs (25).

An *in vitro* study on primary cultures of bovine brain microvessel endothelial cells (BBMEC) showed that neutrophils influence the permeability of the BBB (26). In this study, increase of permeability of BBB was observed and confirmed to be induced by infiltrated neutrophils throun an increase in intracellular Ca^2+^.

However, the transient gathering of neutrophils in the infarct lesion after ischemic stroke remains controversial. A study using endothelin-1-induced cerebral ischemia in rats (ET-1 model) showed that infiltrated neutrophils are phagocytized by macrophages in the first 3 days after stroke onset, but MPO activity keeps increasing, suggesting that MPO may not be the best measurement for neutrophil accumulation (27). But as endothelin-1 has also been found on neurons in the brain out of endothelial cells (28), and it is reported to probably prompt growth of astrocytes after spinal cord injury (29), results using ET-1 models may not be completely credible (30).

### Lymphocytes

Both innate and adaptive immune cells contribute to the inflammatory response after cerebral ischemia. In mice MCAO models, lymphocytes accumulate in the infarct lesion in the first 4 h after ischemia, and depletion of lymphocytes leads to a smaller infarct volume (5, 31). However, the roles of specific lymphocyte subpopulations in the process of inflammatory reaction after cerebral ischemic injury were unclear until recently.

#### T and B Lymphocytes in Cerebral Ischemia

CD4^+^ and CD8^+^ T cells interact with each other. Lower IL-16 expression was observed in CD8-deficient mice in parallel with decreased CD4^+^ T-cell recruitment (32). There were reports about T cell involvement in ischemia/reperfusion (I/R) injury in other organs including the intestine, kidney, and liver. From the results a hypothesis was proposed that T cells may also play a role in I/R injury in the brain. However, as earlier studies mainly focused on monocytes, T cells have been neglected for a long time (33). In 2006, Yilmaz et al. elucidated the contribution of CD4^+^ and CD8^+^ T lymphocytes to the inflammatory and thrombogenic responses in an experimental stroke model. The team discovered that in the first 24 h after ischemic stroke onset, T cell depletion significantly reduced infarct volumes, but lacking B cells did not influence ischemic stroke outcomes. According to their results, both CD4^+^ and CD8^+^ T cells exert detrimental effects on post-ischemic cerebral immune responses (5). Considerable evidence demonstrates the detrimental effects of T cells. Depletion experiments showed improvement of infarction (31), and cytotoxic T lymphocytes have a direct cytotoxic effect on cerebral post-ischemic injuries via the perforin-mediated pathway (34).

T cells are regulated by various cytokines. In an early study, IL-15 was reported to enhance the *in vivo* function of reactive CD8^+^ T cells (35). Later, the effect of IL-15 on CD8^+^ T cells was further characterized (36). Astrocytes, the main source of IL-15 in the brain, have been shown to modulate polarization of CD4^+^ T cells into Th1 cells and support Treg production in co-culture cell conditions. These results provide additional evidence that the central nervous system (CNS) environment affects T cells (37). In later studies, IL-15 was confirmed to be a positive regulator that induces and enhances the Th1 response in the post-I/R cerebral immune response. Lee et al. found that a neutralizing IL-15 antibody likely penetrated that BBB and significantly reduced responses mediated by T cells and natural killer (NK) cells, implying that IL-15 could be a novel treatment target after cerebral I/R (38).

IL-2 secreted by T cells is one of the cytokines that supports T cell survival (39). Both IL-15 and IL-2 regulate CD8^+^ T cell proliferation *in vitro*, but only IL-15 has an effect on CD8^+^ T cells *in vivo*. Generally, IL-2 levels *in vivo* are too low to regulate CD8^+^ T cell proliferation, but CD4^+^ T cells respond well to this low level (40–42). IL-2 was also found to promote regulatory T cell (Treg) production (42). In experimental autoimmune encephalomyelitis, IL-2 also influences the behavior of NK cells. NK cells also suppress Th17 transcription factors via microglia, and complexes of IL-2 and IL-2 monoclonal antibody reduce Th17 production by CD4^+^ T cells in the CNS. These results may suggest that IL-2 regulates NK cells in CNS immune responses and probably influence post-ischemia immune responses (43).

Targeting B cells in experimental stroke does not influence infarct volume, evolution, or cerebral blood flow during the acute phase (44, 45). However, some findings indicate that B cells are beneficial lymphocytes in the immune response after cerebral ischemia. B cells exert neuroprotective effects in the process, and there is ample evidence that B cells are the main regulatory immunological cells in the inflammatory process after ischemic brain injury (45–47). B cells have a probable protective function; they were observed to limit infarct volume and functional neurological deficits by inhibiting activation and recruitment of other immune cells including T cells, macrophages, and microglia into infarct lesions during post-ischemia responses. B cells are believed to promote the recovery process and are regarded as a potential therapeutic target for neurological function recovery after ischemic cerebral injury (47). However, other evidence suggests that B cells may hamper long-term recovery and are probably responsible for delayed cognitive impairment after ischemic stroke. In a mouse distal MCAO model, activated B cells infiltrated infarcted lesions weeks after stroke, and mice developed delayed deficits in long-term potentiation and cognition that could be prevented by an anti-CD20 antibody (48).

The influence of B cells on cerebral ischemia outcomes remains controversial. However, whether B cells display duel function or not, they remain a potential therapy target. In order to have a better knowledge on the role B cells play during and after cerebral ischemia, further studies are required to clarify B-cell related short- and long-term prognoses.

#### Regulatory T and B Cells (Tregs and Bregs)

The newly defined subtype of regulatory B cells (49) have protective effects in some autoimmune diseases. They are thought to regulate the behavior of activated B lymphocytes in the immune response after cerebral ischemia. Recently they were confirmed to have a protective effect against brain injury (50). A CD1d^hi^CD5^+^ phenotype of regulatory B cells that secrete high levels of IL-10 (B10 cells), has been found to play a role in contact hypersensitivity responses, but this phenotype accounts for a low proportion of spleen B cells. Depletion of B10 cells in the spleen enhanced inflammatory responses (51). This phenotype exerts regulatory effects during *Listeria* infection. In animal models, depletion of B10 cells enhanced bacteria clearance, which was accompanied by significantly more macrophage phagocytosis of bacteria, even if macrophages were re-stimulated in an external environment. Collectively these results indicate that B10 cells may have a negative regulatory effect on macrophage cytokine production.

B10 cells were also found to shape the responses in BALB/c mice infected with *Leishmania major* through IL-10 production (52). These results underscore the need for research into the regulation pathways of IL-10 secreted by regulatory B cells (53). Offner and Hurn first implicated B10 cells as a major regulatory subtype in the post-ischemic immune process. They pointed out that this subpopulation may be a potential target of stroke treatments (45). B10 cells accumulate in the infarct area 48 h after 60-min MCAO. Although only a small amount of B10 cells were found in the striatum, they were the main regulatory factors in post-ischemic responses compared to intraperitoneal B cells. Moreover, smaller cortical infarct volumes were observed in mice injected with IL-10-competent B cells compared to those injected with intraperitoneal-derived B cells (46).

Regulatory B cells affect multiple pathways, one of which is via suppressing pro-inflammatory T cells and enhancing regulatory T cell expansion (50, 54, 55). IL-10 secreted by B10 cells plays a role in polarizing the Th cell response toward the Th2 phenotype. Researchers proposed that the process was regulated by IL-12 (52). Tregs are a CD25^+^ Foxp3^+^ cell subset that only account for 10% of peripheral CD4^+^ T cells (32). They play an active regulatory role in the post-ischemic brain (6) and were identified as key mediators of ischemic stroke. Tregs possess an intrinsic propensity for migration, which increases the likelihood of Treg-endothelial cell interaction. Moreover, Treg-mediated infarction development can be prevented by platelet depletion, indicating that the Treg-related immune process is closely related to thromboembolism (56). Numerous studies have shown that Tregs have positive effects in the brain. Some authors have reported that Tregs down-regulate immune cell infiltration, which reduces inflammatory reaction and thus protects neurons. Together with monocytes, Tregs also promote neovascularization after ischemic stroke (32). Tregs function by promoting the production of various cytokines such as TGF-β, IL-10, and IL-35.

Tregs are mediated by neural cell-specific genes such as the serotonin receptor (Htr7) and respond to serotonin, and they are also sensitive to selective serotonin reuptake inhibitors (SSRIs) (32). There is no evidence that the role T cells play in post-ischemia cerebral injury is influenced by antigen recognition, T cell receptor (TCR) stimulatory pathways, or thrombus formation. It is an antigen-independent detrimental effect that T cells exert on the process (33). Tregs are crucial for promoting post-ischemic immunosuppression, which provides a partial neuroprotective effect but also increases the risk of post-stroke infection (32).

#### γδT Cells

Integrins and interleukins play significant roles in assisting immune cells in post-ischemia immune responses. They influence inflammation development and usually aggravate neurological and functional outcomes after ischemic brain injuries. Gamma delta T cells (γδT cells) produce integrins and interleukins.

T cells infiltrate into the brain where they promote infarction evolution and subsequent neurological deficits via IL-23 and IL-17. The ILs mainly derive from γδT cells instead of the long-believed CD4^+^ Th cells (57). The pivotal role of IL-17-secreting γδT cells was recently demonstrated, and the CC chemokine receptor 6 (CCR6) was found to be a primary regulator. In CCR6^−/−^ MCAO mice there was decreased accumulation of IL-17-producing γδT cells (nTγδ17 cells) accumulation, as well as smaller infarct volumes due to less IL-17-dependent induction of the CXC chemokine and neutrophil infiltration (58). Zheng et al. found that IL-23 stimulates production of other cytokines and the transcription factor forkhead box P3 (Foxp3) in cerebral ischemia. They also showed that RNA interference knockdown of IL-23p19 prevented cerebral ischemic injury by reducing inflammation after stroke onset. IL-23 deficiency enhanced interferon (IFN)-γ and Foxp3 expression levels in delayed cerebral ischemic mice, and IL-17, IL-4, IFN-γ, and Foxp3^+^ cells were found in the ischemic hemisphere (59).

Systemic treatment with IL-33 showed beneficial effects against stroke (60). It promotes Th2-type effects and thus increases the plasma level of Th2-type cytokines and fewer pro-inflammatory microglia/macrophages in the infarct lesion (61). IL-33 also promotes the infiltration of NK cells, reduces activated glial cells, and increases IL-10-expressing Tregs. However, MCAO mice treated with IL-33 were susceptible to serious lung infection, which implies that IL-33 may exert a dual effect on the prognosis of ischemic cerebral injury. The data suggest that IL-33 might be a target treatment when given under close surveillance for infection or in combination with antibiotics (62). Xiao et al. reported another effect of IL-33 after ischemic brain damage. They studied the modulation effect of IL-33 of splenic T cell responses after cerebral ischemic stroke and found that intraperitoneal IL-33 pre-treatment reduced neurological deficit scores and infarct volumes after 30 min of MCAO, decreased IFN-γ^+^ T cells, and increased Foxp3^+^ T cells in the spleen. IL-33 pre-treatment also reduced IFN-γ production and mRNA levels of the transcription factor T-bet, but increased IL-4, IL-10, TGF-β, GATA-3, and Foxp3 in the spleen. Xiao et al. proposed that IL-33 participates in the post-ischemic immune responses by inhibiting the Th1 response and promoting the Treg response and therefore will be a potential ischemic stroke treatment (63).

#### NK Cells

NK cells are innate lymphocytes. They are rapidly mobilized and recruited during the super acute phase of immune responses (64, 65). Gan et al. showed that NK cells are recruited only 3 h after ischemia and peak at day 3 (66). The temporal dynamics of immune cell accumulation in a temporal MCAO model were observed, but there is no report regarding an increased number of NK cells in the ischemic hemisphere (67). According to Zhou et al., the behavior of NK cells is related to the time of ischemia during a stroke. Their research showed that invading NK cells accumulate in ischemic lesions but do not differ between mice with 30-min partial MCAO and 90-min total MCAO. However, the authors only assessed cytokine expression at a single subacute time point, which should be extended and further tracked in future studies (68).

Later studies suggested that NK cells may indeed influence ischemic brain injury outcomes. NK cell recruitment in the early stage of ischemic stroke is required in the Th1 response priming of CD4^+^ T cells by IFN-γ (69). NK cells also influence the level of activated CD8^+^ T cells by killing recently activated CD8^+^ T cells in an natural killer group 2D (NKG2D)- and perforin-dependent manner, and thus influence cellular immune responses (70). Gan et al. found that in the human brain, NK cells infiltrate into peri-infarct areas or the ischemic hemisphere. They help catalyze neuronal death via the perforin/granzyme apoptosis pathway, directly or indirectly co-effecting with immigrant cells or brain-resident cells, thus accelerating ischemic infarction (66). Zhang et al. found that NK cells promote the process via IFN-γ and that NK cells are dose-dependently affected by IP-10 via CXCR3. NK cells are related to disintegration of the BBB, and this injury process was aggravated by IP-10. In their study, the number of NK cells peaked 12 h after ischemic stroke onset (71).

NK cells kill inactivated resting microglia via NKG2D (CD314) and NKp46, one of the natural cytotoxicity receptors (NCRs). Activated NK cells rapidly form immunological synapses with microglia and mediate perforin polarization at the interface between NK cells and microglia (72). NK cells also closely contact astrocytes. IL-15 is one of the extrinsic signals that regulates the development and maturation of NK cells (73–75). Previous studies have demonstrated how IL-15 influences the development of mature NK cells (76), but more recent research confirmed that IL-15 is produced by glial cells, and the level is determined by glial cell activity. Reactive glial cells, mainly astrocytes, express IL-15 in the acute phase of CNS inflammation, but in a study, microglia were recruited as the minor source of cytokines (77). Later studies showed that in the glial fibrillary acidic protein (GFAP) promoter-controlled IL-15–expressing transgenic mouse (GFAP-IL-15^tg^) line, mice that express astrocyte-derived IL-15, NK cells, and CD8^+^ T cells increasingly accumulate in post-reperfusion responses. The results suggested that astrocyte-derived IL-15 might recruit NK cells and CD8^+^ T cells in post-ischemic cerebral reactions, exacerbating the infarction and subsequent neurological deficits (36).

### Leukocyte-Endothelial Cell Interactions and Leukocyte Infiltration

**Figure 1:**
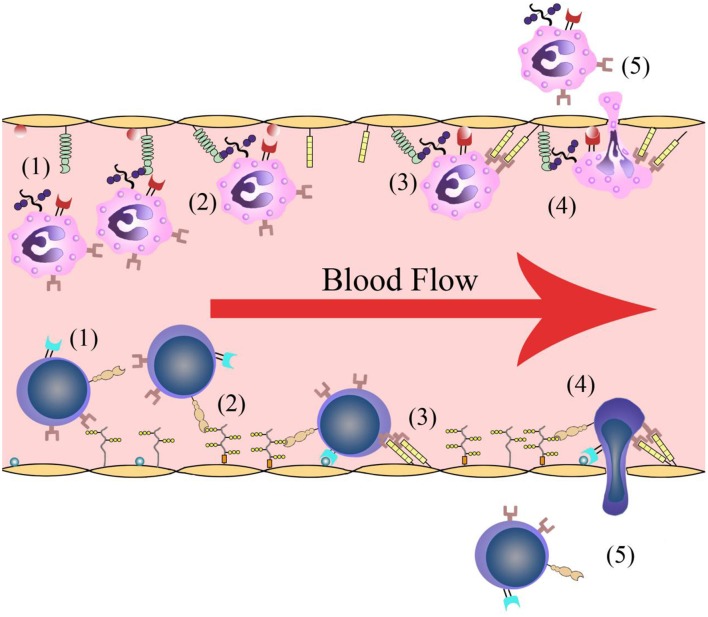
Leukocyte infiltration process. (1) Leukocytes roll along the vascular endothelium. (2) Leukocytes are recognized and initially bind to the blood vessel walls. (3) Leukocyte activation and leukocyte-endothelial cell adhesion. (4) Leukocytes travel through the vascular wall. (5) Leukocytes transmigrate into the ischemic region.

Leukocyte infiltration is crucial in cerebral post-ischemic immune responses. Recruitment and infiltration of leukocytes are dependent on vascular endothelial cells. In the first several hours after stroke onset, leukocytes are rapidly recruited into microvessels in the ischemic region. The recruitment process requires some events including leukocytes rolling along the vascular endothelium and initially binding to the blood vessel walls, followed by leukocyte activation, leukocyte-endothelial cell adhesion, leukocytes traveling through the blood vessel walls, and transmigration into the inflammatory region (Figure 1). Adhesion molecules and cytokines are the main molecules that interact with leukocytes on the endothelial cell membrane. There are changes in microvessels after ischemic stroke onset. The membranes of endothelial cell contain adhesion molecules and regulatory cytokines that interact with activating leukocytes and platelets, which together initiate and participate in thrombosis (9).

#### Adhesion Molecule Expression

Adhesion molecules including integrins, immunoglobulin superfamily members, and selectins induce cell-to-cell or cell-to ECM binding via receptor-ligand formation. Binding is a key stage in cell infiltration and migration and is significant in the early stages of cerebral post-ischemic immune responses.

Among immunoglobulin superfamily members, myeloperoxidase, intracellular adhesion molecule-1 (ICAM-1), and vascular cell adhesion molecule-1 (VCAM-1) have been studied the most with regard to their effects on leukocyte-endothelial cell interaction (78). ICAM-1 is associated with leukocyte accumulation in the ischemic region in the first 24 h, and ICAM-1 levels peak at 12–48 h post-ischemia (79, 80). As is shown in many deficiency/inhibition studies, ICAM-1 depletion reduces leukocyte infiltration and infarct volumes (81). However, a clinical trial using enlimomab, an anti-ICAM-1 antibody claimed that anti-ICAM-1 treatment may bring opposite results. Patients treated with enlimomab obtained worse outcomes (82). VCAM-1 levels increase in post-stroke responses, and the levels are even higher in patients with polyvascular atherothrombotic diseases (83). Evidence suggests a probable beneficial effect of VCAM-1 depletion, suggesting that protein has detrimental effects (84, 85).

Selectins (E-, L-, and P-selectins) separately modulate the recognition and initial binding to vascular endothelial cells and different leukocyte subpopulations. One study reported regulation effects of selectins in cerebral post-ischemic immune responses. P-selectin, which is essential to monocyte-endothelial cell adhesion (86), was associated with BBB breakdown (87, 88). P-selectin continually rise in the acute phase, and the increase persists into the subacute phase, while E-selectin levels start to decline at the end of acute phase (89). However, a study of L-selectin inhibition did not report significant differences (90). This may be related to the specific subpopulations that certain selectins recognize.

The integrin families mainly induce cell-ECM adhesion. The leukocyte function-associated antigen (LFA) group and very late adhesion molecule (VLA) group are distributed on the leukocyte membrane, while glycoprotein groups are found on the membranes of platelets, endothelial cells, and megakaryocytes. LFA-1, macrophate-1 (Mac-1), and VLA-4 mediate leukocyte binding to the activated endothelium in ischemic regions (91). Deficiency experiments of LFA-1 and Mac-1 both showed decreased leukocyte infiltration 24 h after stroke, indicating that both LFA-1 and Mac-1 correlate with brain injury and blood cell-vessel wall interactions after cerebral ischemic injury (92).

#### Cytokine Expression

Cytokines including IL, colony-stimulating factor (CSF), IFN, TNF-α, growth factor (GF), and other chemokine families are crucial mediators in most regulatory pathways. Many cytokines have been found to regulate leukocyte infiltration; for example, TNF-α increases lymphocyte infiltration into interstitial tissues in immune responses to kidney infection (93). IL-1 was found to reduce neutrophil infiltration after liver ischemia (94). Cytokines regulate leukocyte infiltration and affect cerebral post-ischemic responses in many other aspects, as described below.

## Resident Glial Cells and Infiltrating Macrophages/Monocytes

Both resident microglia and bone marrow-derived monocytes may be the source of macrophages in the damaged tissue after cerebral ischemia (95). As the key immune cells in acute and subacute stages after cerebral ischemia, resident microglia and infiltrating monocytes share certain features, while differ in some other aspects, which lead to plenty of comparative studies on the two cell populations.

Three murine cell culture models, namely iPSC microglia, iPSC-macrophages and NSC-primed iPSC-macrophages were cultured to elucidate pro-inflammatory and anti-inflammatory features of brain resident and infiltrating monocytes during cerebral ischemia. Consistency of results *in vitro* and *in vivo* suggested that iPSC-derived neuro-immune cell culture models can be possibly useful in related researches (96).

### Resident Glial Cells

Resident microglia are rapidly activated in the first 24 h after transient MCAO in mice. Schilling et al. used green-fluorescent protein bone marrow chimeric to show that resident microglia were the main source of macrophages in infarcted area (95). Glial cells are deeply involved in the acute phase of the post-ischemic inflammatory process. Microglia depletion was reported to increase the infiltration of neutrophils, macrophages, and CD4^+^ T, and NK cells in the brain and reduce the accounts of subtypes of these splenic leukocytes (97). Microglia confer a protective effect against the post-ischemic immune process via their inhibition of astrocytes; they are crucial for neuron-astrocyte crosstalk that occurs in immune responses following cerebral ischemia (68).

Microglia up-regulate expression of triggering receptor expressed on myeloid cells 1 (TREM-1), which prompts microglial M1 polarization and neutrophil recruitment by increasing mRNA levels of M1 markers and chemokines, as well as protein levels of ICAM-1. Microglia TREM-1 regulates cerebral post-ischemic immune responses by activating downstream pro-inflammatory pathways through interaction with spleen tyrosine kinase (SYK), and the final effect leads to increases in inflammatory cytokine levels, chemokine production, and pyroptosis (98). Activated glial cells produce a large amount of inflammatory cytokines including IL-1β, IL-6, TNF-α, and nitric oxide. All of these cytokines promote the evolution of inflammation, and therefore lead to poor outcomes following ischemic cerebral injury (68, 99–101). An *in vitro* model of microglia activation was established to study the delayed inflammation after cerebral ischemia. By exposing microglia to oxygen glucose-deprived (OGD) neurons and astrocytes, an ischemia-like microenvironment is developed (102). The study reported that microglia are activated by OGD and induce neuron death with TNF-α and thus contributes to delayed inflammation (103).

Microglia are also one of the two cellular sources of induction of TNF receptor associated factor 2 (TRAF2); the other source is neurons. Li et al. discovered TRAF2 is induced after ischemic stroke, and this may inhibit necroptosis by inhibiting the association between receptor interacting protein 3 (RIP3) and mixed lineage kinase domain-like (MLKL), the activation of which contributes to necroptosis. Thus, the authors regarded TRAF2 as a novel regulator of cerebral ischemic injury (104). The results indicate that microglia participate in post-ischemic immune responses via TRAF2 production. Microglia highly express danger-associated molecular pattern molecules (DAMPs), co-effecting with purines to induce the expression of pro-inflammatory molecules in infiltrating leukocytes, and they also prime dendritic cells for antigen presentation (91). Glial cells are also mediated by chemokines. Chemokine-liked factor 1 (CKLF1) was found to increase expression of pro-inflammatory cytokines and decrease that of anti-inflammatory cytokines in ischemic lesions. CKLF1 was also found to modulate microglia/macrophages toward an M1 phenotypic polarization, thus aggravating the inflammatory response and contributing to a poor prognosis following ischemic stroke (105–107).

Several studies have confirmed that sphingosine 1-phosphate receptor subtype-1 (S1P1) and S1P3 are related to microglial activation (108–110). M1 polarization is activated by S1P3 mainly in activated microglial (109). Receptor-mediated S1P signaling in regulation of inflammatory cells has been described by multiple groups (111, 112). Expression of S1P receptor-1 (S1PR1) by lymphocytes was found to play a role in lymphocyte egress from the thymus and secondary lymphoid tissue or organs. T cells tend to migrate toward S1P1 in the first stage after activation of the inflammatory response, but the ability disappears after day 1. However, after 72 h, T cells recover S1P1 mRNA and regain their responses to S1P1 (113). This could be a possible explanation for brain T cell accumulation in the first 24 h after ischemic stroke. S1P is also reported to play a role in pro-inflammatory responses (114–116), mainly in activated microglia (115–117). One of the drugs targeting S1PR1, FTY720 (Fingolimod), has protective activity in different animal models of transplant and autoimmune diseases and is therefore used in experiments as an immuno-suppressant (114). It was also observed to suppress neuronal damage and microglial activation in rodent models of cerebral ischemia (118). A proof-of-concept trial shows that in patients with acute ischemic cerebral stroke, FTY720 treatment within 72 h of stroke onset brings better outcome (119). In this trial, FRY720 is reported to have reduced secondary tissue damage and neurological deficits, as well as prompted functional recovery. Other experiments showed that lipopolysaccharide (LPS) stimulation reduced microglial expression of S1P2, S1P4, and S1P5, suggesting the down-regulation of S1PR subtypes may also contribute to the persistence of microglial activation during inflammatory conditions, given that the receptor for fractalkine, a negative regulator of microglial activation, was down-regulated by LPS stimulation of microglia from aged mice (120). NK cells are also regulated by S1PRs. S1PR5 has been shown to be necessary for NK cell egress from lymph nodes and bone marrow, suggesting that behavior of NK cells may be in part mediated by S1P and S1PRs (121). Gaire et al. found that S1P1 regulates M1/M2 polarization. Specifically, S1P1 activation influences mitogen-activated protein kinases and phosphoinositide 3-kinase/Akt activation in the ischemic hemisphere, and those pathways can increase M1 polarization while simultaneously decreasing M2 polarization. The influence is especially significant on microglia M1 polarization (122). These results may lead to further study of S1P subtypes on their relationships with and regulation of the M1/M2 polarization of microglia. S1P also activates astrocytes. Local microinjection of S1P exacerbated cerebral infarction in MCAO models, and the microglia/macrophage-specific marker Iba1 and astrocyte-specific marker GFAP both significantly increased (114). This supports the hypothesis that microglia and astrocyte activation is detrimental to recovery from cerebral ischemia.

### Infiltrating Macrophages/Monocytes

Macrophages/monocytes mainly accumulate in the ischemic region in the subacute phase. Levels peak in the infarct territory 72–96 h after ischemic induction (7).

Increasing invading macrophages usually suggests a better prognosis after cerebral ischemia. It has been reported that low lymphocyte-to-monocyte ratio is an independent risk factor for poor outcome in acute cerebral ischemic stroke cases treated with thrombolytic therapy (123).

Neutrophils are found to promote monocyte recruitment (13) via proteins released from neutrophil granules (124). CD14^+^ monocyte differentiation induces polarization of CD4^+^ T cells into Th-17 cells during migration across the BBB (125). The process involves cytokines including TGF-β and granulocyte-macrophage colony-stimulating factor (GM-CSF). Both cytokines function in inflammatory responses. In the CNS, TGF-β expression enhances immune cell infiltration and increases autoimmune damage (126). GM-CSF has been confirmed to promote monocyte differentiation into dendritic cells (125). These cytokines are secreted by various cells including astrocytes and microglia, implying there may be a combination of regulatory effects by various cell types (127, 128).

Different behavior models are also observed during the recovery after ischemia. Infiltrating macrophages tend to massively cluster to infarct area 72 h after stroke onset, while microglia display a migration from infarct center to peri-infarct region during several days after the cerebral ischemic events (129).

Infiltrating monocytes are found to have different molecular signature and electrophysiological properties from resident microglia. The study also covered microglia and monocyte behaviors during the delayed phases after cerebral ischemic injury. Differences in genomic features were also detected in resident microglia and infiltrating monocytes (130).

Differences on inflammatory expression profiles between microglia and invading macrophages are observed in study and are believed to shape repair and pro-regenerative mechanisms after stroke. Expression analysis in pMCAO mice models show a reduction in expression of pro-inflammatory genes, while bone marrow-derived macrophages have an inflammatory phenotype (129).

In a study of periphery immune cell invasion to the cerebral parenchyma after cardiac arrest and resuscitation, researchers found a parallel significant increase of monocytes in the bone marrow and blood, which they think may suggest a strong coupling of peripheral immune response and CNS immunity, providing a potential neuroprotective therapy by targeting the pro- and anti-inflammatory signals in the periphery (131).

Like the way microglia switch between M1 and M2 subtypes in a certain context, it is reported that infiltrated monocytes in the infarct area display different expression feature from those surrounding the infarct area, suggesting that infiltrated monocytes may react specifically to different micro-environments (132).

Unique transcriptomic profiles were detected in both resident microglia and bone marrow-derived macrophages in ischemic hemispheres in tMCAO in rats. Resident microglia are found to mainly display pro-inflammatory phenotype, while infiltrating macrophages recruited in the early stage after cerebral ischemia play anti-inflammatory, phagocytic and wound healing function. These bone marrow-derived macrophages, however, showed a functional shift toward a pro-inflammatory phenotype (133).

In certain context macrophages can be polarized into a state sharing part of signature features of M2 cells, namely the M2-like phenotype (134). Similar switch have been observed in many studies. For example, according to Lidia Garcia-Bonilla and co-workers, after cerebral ischemia, early accumulation of CCR2^+^Ly6C^hi^ monocytes, an pro-inflammatory monocyte subtype is observed in a study. But weeks later a tendency of CX3CR1^+^Ly6C^lo^ subtype accumulation is detected, which are believed to be switched from CCR2^+^Ly6C^hi^ subtype instead of derived from blood borne monocytes (135).

Later a study confirmed that infiltrating monocytes tend to display an M2-like phenotype after stroke, which is neuroprotective. According to genomic analysis, this tendency might be stronger than that of microglia (130).

A study on the role of choroid plexus played in post-ischemic cerebral immune responses found that in cerebrospinal fluid, polarized M2-like monocyte-derived macrophages can migrate into the ischemic hemisphere, and the process improves motor and cognitive prognosis with no influence on infarct volume. The result may provide an alternative novel therapy for cerebral ischemic stroke (136).

Some clinical trials have been conducted targeting microglia/macrophages. An open-label, evaluator-blinded study of minocycline, a deactivator of macrophages showed its favorable effects on patients acute stroke onset when taken orally, probably by inhibiting microglia activation in the acute phase (137). As minocycline is inexpensive and safe with easy access, it makes a pretty promising treatment on ischemic stroke. A later pilot study of a small sample of acute stroke patients showed intravenous minocycline is safe. Meta-analysis of three human trials suggests minocycline may reduce disability after stroke, but larger trials are required to ensure the effect of minocycline in post cerebral ischemia treatment (138).

## Stroke-Induced Immunodepression

Stroke-induced immunodepression (SIID) occurs in both experimental models and clinical cases. SIID is characterized by lymphopenia, up-regulation of anti-inflammatory cytokines, and splenic atrophy (139). There are other factors influencing SIID, such as glucocorticoids, acetylcholine, adrenaline, and noradrenaline (6).

Tregs are mainly involved in immunosuppression via cytokine production (32, 140). One hypothesis posits that the systematic switch to Th2 responses exerts a long-term effect on SIID. A decreased IFNγ/IL-4 ratio is reportedly associated with impaired IFN-γ production, which blockades infection defense (141). IL-4 plays a role in exacerbation of various diseases, and play a robust regulation role in the Th1/Th2 switch (142). Reducing Th1-type inflammation, IL-33 probably aggravates SIID via Th2-promoting effect although the effect reduces infarct volumes. This led to a phenomenon that IL-33 treated animal MCAO models showed more limited infarction and better outcomes as well as worse clinical deficits (61). Many animals died of pneumonia, which shares the highest morbidity in clinical stroke cases (141). These results emphasize the significance of early use of antibiotics after stroke onset.

## Conclusion

Cerebral post-ischemic immune responses involve the CNS; peripheral immune cells; the BBB; vascular endothelial cells; and various inflammatory molecules including cytokines, adhesion molecules, selectins, globulins, and fibrillation. Immune cells including resident glial cells play the most pivotal role in the process. Early recruited immune cells such as neutrophils and T lymphocytes influence ischemic injury in the acute phase, while cells infiltrating the ischemic region in the subacute phase mainly influence neurons and functional remodeling and recovery processes. Various subpopulations of immune cells have been reported to exert dual effects on the evolution of and recovery from ischemic stroke (Table 1). Immune cells influence ischemic stroke mainly via inflammatory factors including cell adhesion molecules, cytokines, and related receptors. Some of these molecules have been identified as therapeutic targets, but their reactions to specific drugs require further study.

**Table 1:** Effects of immune cells in immune responses after cerebral ischemia.

**Immune cell**		**Effects in cerebral post-ischemic responses**
Neutrophils		(1) Promote immune cell recruitment to the ischemic region, including lymphocytes, monocytes, and platelets; (2) Clearance of dead cells, debris, and bacteria as a defense against the increased risk of infection as a result of immunosuppression after stroke; (3) Involved in tissue repair and remodeling processes
T lymphocytes		(1) Infiltrate into infarct areas to promote ischemic injury via IL-17, IL-23, and IL-33 secreted by γδT cells; (2) Produce IL-2 to reduce Th17 production by CD4^+^ T cells
Regulatory T cells		(1) Reduce immune cell infiltration; (2) Promote neovascularization; (3) Promote immunosuppression
B lymphocytes		(1) Inhibit the activation and recruitment of other immune cells; (2) Promote the recovery process; (3) Harmful to long-term recovery and probably lead to delayed neurological and cognitive function deficits.
Regulatory B cells		(1) Down-regulate macrophage cytokine production; (2) Suppress pro-inflammatory T cells and enhance the expansion of regulatory T cells; (3) Promote Th2 polarization via IL-12
NK cells		(1) Participate in Th1 priming of CD4+ T cells; (2) Kill recently activated CD8^+^ T cells in an NKG2D- and perforin-dependent manner; (3) Promote neuronal death
Astrocytes		(1) Produce IL-15, mediating Th1 polarization of CD4^+^ T cells, enhancing production of Tregs, and influencing maturation of NK cells; (2) Activation aggravates ischemic injury
Macrophages /microglia	Residental microglia	(1) Prompt microglial M1 polarization and neutrophil recruitment; (2) Induce neuron death through TNF-α; (3) Migrate from infarct center to peri-infarct regions in delayed phase
	Infiltrated macrophages	(1) CD14^+^ differentiation induces CD4^+^ T cell polarization into Th-17 cells when migrating across the BBB via TGF-β and GM-CSF; (2) Display anti-inflammatory, phagocytic and wound healing function in early phase; (3) Migrate from infarct center and tend to display an M2-like phenotype in delayed phases

## Author Contributions

YW and AS drafted the manuscript. JZ, AS, and JS reviewed and modified the manuscript. All authors agreed on the final version.

### Conflict of Interest

The authors declare that the research was conducted in the absence of any commercial or financial relationships that could be construed as a potential conflict of interest.
